# Applied mediation analyses: a review and tutorial

**DOI:** 10.4178/epih.e2017035

**Published:** 2017-08-06

**Authors:** Theis Lange, Kim Wadt Hansen, Rikke Sørensen, Søren Galatius

**Affiliations:** 1 Department of Biostatistics, University of Copenhagen, Copenhagen, Denmark; 2 Center for Statistical Science, Peking University, Copenhagen, Denmark; 3 Department of Cardiology, University Hospital Bispebjerg, Copenhagen, Denmark; 4 Department of Cardiology, Rigshospitalet, Copenhagen, Denmark

**Keywords:** Mediation analysis, Causal inference, Pathway analysis, Acute coronary syndrome, Randomized clinical trials

## Abstract

In recent years, mediation analysis has emerged as a powerful tool to disentangle causal pathways from an exposure/treatment to clinically relevant outcomes. Mediation analysis has been applied in scientific fields as diverse as labour market relations and randomized clinical trials of heart disease treatments. In parallel to these applications, the underlying mathematical theory and computer tools have been refined. This combined review and tutorial will introduce the reader to modern mediation analysis including: the mathematical framework; required assumptions; and software implementation in the R package medflex. All results are illustrated using a recent study on the causal pathways stemming from the early invasive treatment of acute coronary syndrome, for which the rich Danish population registers allow us to follow patients’ medication use and more after being discharged from hospital.

## INTRODUCTION

As any statistician collaborating with applied researchers (be they doctors, psychologists, or social scientists) knows, once a cause-and-effect relationship has been established, the next point on the agenda is very often “How does this effect come about? What are the underlying mechanisms?” Indeed, such questions have been driving science forward for centuries. Mediation analysis in the general meaning of the term refers to a collection of tools and ways of thinking designed to help applied researchers identify, formalize, and quantify possible mechanisms (i.e., causal pathways) linking a cause to an effect. To name just one example, the search for the mechanisms linking exposure to contaminations with subsequent disease, which was already under way in the 16th century, culminating in Louis Pasteur’s identification of bacteria as the “mediating factor,” was based on reasoning about mediation.

In contrast, statistical mediation analysis, which will be the object of interest in this tutorial, is concerned with quantifying specific causal pathways described by one or more measurements of specific variables that are either assumed or have been shown to be affected by the exposure and themselves affect the outcome. Statistical mediation analysis is broadly said to have been initiated in the seminal 1986 paper by Baron & Kenny [1]. As will be demonstrated in this tutorial, one of the main contributions of statistical mediation analysis is to translate the loose or intuitive concepts of, for example, Pasteur’s “mediating factors” into statements expressed as statistical models using mathematical formalism. Another important contribution, which will also be thoroughly discussed in this tutorial, is the derivation of the assumptions that must be satisfied before causal pathways can meaningfully be estimated from data.

For the remainder of this tutorial, mediation analysis will be taken to mean statistical mediation analysis only. The reader is expected to be familiar with statistical modelling and inference, as well as the distinctions between statistical associations and causal effects (i.e., why observational studies are harder to interpret than randomized studies). A prior knowledge of theoretical causal inference in general, or mediation analysis in particular, is neither assumed nor required.

The rest of this tutorial is structured as follows. Section 1 introduces the case that will be used to illustrate theoretical concepts throughout. In section 2, the mathematical framework for mediation analysis is introduced and the required assumptions are presented and discussed. Methods for estimation in real-life settings are presented in section 3, and the tools applied to the illustrative case are described in section 4. Finally, some current methodological challenges within mediation analysis (in particular, sensitivity analyses and multiple mediators) are discussed in section 5. Due to the nature of the illustrative case, special focus will be given to handling complex mediators. The simplementation will largely build on the recently released R package medflex [2].

## SECTION 1: AN ILLUSTRATIVE CASE

Acute coronary syndrome (ACS) presents as a cardiac emergency caused by sudden obstruction of a coronary artery, most frequently due to thrombus formation in an existing atherosclerotic lesion in the vessel wall. In the acute phase, treatment aims to prevent sudden cardiac death and complications by halting the progression of thrombus formation, managing symptoms, and identifying and treating coronary obstructions; the latter goal involves early cardiac catheterization. Once stabilized, patients receive secondary preventive medication and undergo risk factor modification to prevent future cardiovascular events, including death.

Using Danish register data, we have previously established [3]that in a population of patients with a first hospitalization for ACS, the use of an early invasive treatment strategy was associated with a lower short-term risk of cardiac death and readmission for myocardial infarction than a conservative approach. It has been speculated that some or, in selected subgroups, all of the long-term benefit provided by an invasive treatment strategy is mediated through better secondary preventive medical therapy. In this case study, we will explore the relationship between an early invasive treatment strategy, secondary preventive medication, and death from all causes.

Following previous research and current guidelines [4], we define an early invasive strategy as cardiac catheterization performed within 72 hours of index hospitalization, thus assuming an intention to treat with reperfusion therapy, if appropriate based on coronary anatomy. In contrast, we define a conservative approach to be when an angiographic assessment was performed more than 72 hours after the index hospitalization or not at all.

The general recommendations for secondary preventive medical therapy in the Danish national guidelines for treating ACS include: lifelong aspirin, a P2Y12 receptor inhibitor (clopidogrel, prasugrel, or ticagrelor) for 12 months, lifelong statin therapy, and treatment with a β-blocker for at least 2 years. We defined a person as adhering to secondary medication for a given drug if a prescription was filled within 30 days of discharge or if the patient was in possession of a sufficient quantity of the drug to cover the initial 30 days after discharge (see Hansen et al. [5] for further references). As the recommended secondary mediation includes 4 drugs, we have 4 variables that may function as potential mediators. The outcome was death from all causes during follow-up. As this was an observational study, we attempted to control confounding by including information on age, sex, calendar year, net household income, educational level, cohabitation status, myocardial infarction, cardiac arrhythmia, heart failure, pulmonary oedema, cardiogenic shock, valvular heart disease, cerebrovascular disease, cancer, chronic obstructive pulmonary disease, diabetes with complications, acute and chronic renal failure, sepsis, pneumonia, anaemia, respiratory insufficiency, prior revascularization, prior in-hospital bleeding, and the use of antihypertensive medications, aspirin, lipid-lowering drugs, vitamin K antagonists, glucose-lowering drugs, loop diuretics, or chronic obstructive pulmonary disease medication.

As all Danes are given a unique identification number at birth, which is recorded in all subsequent dealings with the health care system, we were able to identify all patients hospitalized with a first ACS. We excluded patient who were discharged on the day of admission to ensure that there had indeed been time to conduct proper electrocardiographic monitoring and sequential measurements of cardiac troponins. Only patients aged 30-90 years were included. In patients younger than 30 years, it was assumed that atherosclerosis may not be have been the predominant underlying cause of ACS; similarly, patients older than 90 years were excluded, as they were deemed too frail for invasive procedures. Finally, we restricted the study cohort to those patients who had not died or emigrated within 30 days of discharge. We were forced to use the condition of 30-day survival to ensure that patients actually had the time to initiate secondary therapy. This shortcoming is discussed further in section 5.

A total of 49,640 patients (mean age, 66.3 years; standard deviation, 12.8 years; 35% females; 83% myocardial infarction) were included. Forty-six percent had received an early invasive treatment strategy. The number of deaths during follow-up (median: 3.6 years) was 10,847 (21.9%). Concomitant use of all 4 drugs (aspirin, a P2Y12 receptor inhibitor, statin, and β-blocker) after discharge was observed in 56% of patients (68 vs. 45% in the early and conservative invasive groups, respectively). Receiving an early invasive treatment strategy was associated with a lower incidence rate of all-cause death (3.1 vs. 8.1 deaths per 100 person-years; adjusted hazard ratio [HR], 0.71; 95% confidence interval [CI], 0.67 to 0.74; p<0.001) compared to a conservative approach.

This case will be used throughout this tutorial. It should be stressed that the analyses presented in this tutorial are meant as a pedagogical tool for explaining mediation analyses. Accordingly, a full discussion of the medical implications as well as some case-specific limitations are not included. The interested reader is referred to Hansen et al. [5] for an in-depth discussion.

## SECTION 2: THE MATHEMATICAL FRAMEWORK FOR MEDIATION ANALYSIS

**Figure 1.**
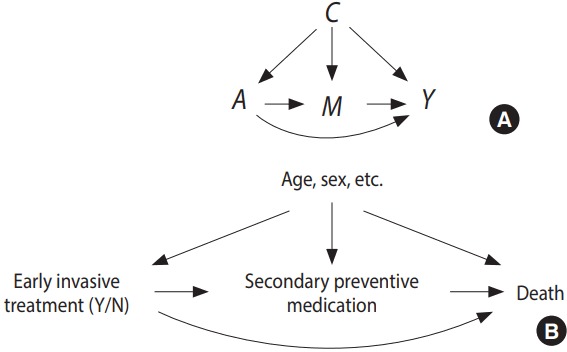
Generic directed acyclic graph for mediation analysis (A) and for the illustrative example (B). C, confounder; A, exposure; M, mediator; Y, outcome.

The first step of any mediation analysis is to describe pre-existing beliefs about the causal structure in which the mediation analysis is to be conducted. Directed acyclic graphs (DAGs) are the method of choice for doing so. An example is given in Figure 1, where the assumed causal structure of the illustrative case is presented. An arrow in a DAG implies that we believe a possible causal connection exists between the 2 variables in question. A causal connection, such as *A* → *B*, can loosely be interpreted as: “if we actively change the variable *A*, the distribution of *B* might change.” Note that the real assumption in the DAG *A* → *B* is that an intervention on *B* will not change *A*. For a more detailed introduction to DAGs see Pearl [6]. The defining feature of a mediator is that it is positioned between the exposure and the outcome when following the directions of the arrows in the DAG. The DAG must also include all likely common causes of any pair constructed from an exposure, mediator, and outcome. In Figure 1, early intervention is assumed to affect the secondary treatment strategy (here defined as drug initiation within 30 days), which in turn affects mortality; this effect is called an indirect effect. There might also be other mechanisms, not involving the secondary treatment strategy, through which early intervention can affect mortality. These are subsumed within the direct arrow from early invasive strategy to death, which therefore can be thought of as mediation through all mediators except for the secondary treatment strategy; this is called a direct effect.

From an intuitive point of view, mediation analysis boils down to describing what would happen if a) the indirect pathway was the only causal pathway between exposure and outcome and b) the indirect pathway could be deactivated completely. However, this intuition is not sufficient to mathematically define the corresponding parameters to be estimated. We therefore introduce so-called counterfactual variables [6]. Building on the variables defined in Figure 1, these are:

•*Y_i_* (*a, m*) is the outcome achieved for person *i* if, possibly contrary to fact, exposure had been set to a and mediator to *m*.
•*M_i_* (*a*) is the mediator achieved for person *i* if, possibly contrary to fact, exposure had been set to *a*.

The subscript *i* will be omitted when referring to a randomly picked person. The counterfactual variable *Y_i_* (1, *m*) corresponds to the death time observed in a double-intervention randomized trial where early intervention had been used and secondary medication set to *m*. Likewise, the counterfactual variable *M_i_* (1) is the secondary medication observed in a single-intervention randomized trial where early intervention had been used. One can combine the two counterfactuals, yielding so-called nested counterfactuals defined as *Y* (a, M (a^*^)). When *a*=a^*^, the nested counterfactual simply corresponds to the observations one would observe if early intervention had been set to *a*. In the mediation analysis literature, the effect one would observe in a simple randomized trial is referred to as the total effect of treatment (i.e., early intervention), and it is defined as a comparison of the distribution of *Y* (1, *M* (1)) with that of *Y* (0, *M* (0)). The comparison could be done as a comparison of average values, but with a survival outcome, it would be more common to compare the 2 arms of the trial using a Cox model, leading to a causal HR quantifying the effect of treatment. The books by Pearl [6] and Hernán & Robins [7] provide a thorough introduction to why 1 arm of a randomized trial can be used to estimate the distribution of the counterfactual variable *Y* (1, *M* (1)), which is a quantity defined for the whole population, not only the people in the *A*=1 arm.

Realizing that the traditional 2-arm randomized controlled trial can be viewed as a double-intervention trial where, for instance, in the *a*=0 arm, treatment is set to 0 and the mediator to the value it would naturally take for that person when treatment is set to 0, leads to the following definition of the so-called natural direct and indirect effects. For ease of presentation alone, we compared the counterfactuals using their average values, but other scales such as odds ratios (ORs) could equally well have been used.

 Total effect of treatment:

  =*E*[*Y* (1, *M* (1))]−*E*[*Y* (0, *M* (0))]

 =(*E*[*Y* (1, *M* (1))]−*E*[*Y* (1, *M* (0))])+(*E*[*Y* (1, *M* (0))]−*E*[*Y* (0, *M* (0))])

 =natural indirect effect + natural direct effect

Written in words, the natural indirect effect is the effect you see by changing the mediator, as if you had changed the treatment without actually changing the treatment itself. Likewise, the natural direct effect is the effect you see by changing the treatment, but keeping the mediator fixed at whatever level it would be had you not changed the treatment. Thus, by introducing the nested counterfactual *E*[*Y* (*a*, *M* (a^*^))] for *a* ≠ a^*^ we can give a precise mathematical definition of mediation. This definition was originally introduced by Pearl [8] and much work has since been published on identification, estimation, and applications, culminating in the recent book by Vanderweele [9], where a comprehensive list of references can be found. As the definition of natural direct and indirect effects at its core builds on comparing distributions of nested counterfactuals, these effects can just as easily be expressed on other scales than the averages. For a survival outcome, it would, for instance, be more common to decompose the HR as follows:

$$\mathrm{HR}\;\mathrm{of}\;a=\;1\;\mathrm{vs}.\;a=\;0\;=\frac{\mathrm{hazard}\;\mathrm{for}\;Y\;(1,\;M\;(1))}{\mathrm{hazard}\;\mathrm{for}\;Y\;(0,\;M\;(0))}\;=\frac{\;\mathrm{hazard}\;\mathrm{for}\;Y\;(1,\;M\;(1))}{\mathrm{hazard}\;\mathrm{for}\;Y\;(1,\;M\;(0))}\times \frac{\mathrm{hazard}\;\mathrm{for}\;Y\;(1,\;M\;(0))}{\mathrm{hazard}\;\mathrm{for}\;Y\;(0,\;M\;(0))}\;=\mathrm{natural}\;\mathrm{indirect}\;\mathrm{HR}\times \;\mathrm{natural}\;\mathrm{direct}\;\mathrm{HR}$$

From the derivations above, it is apparent that the key to employing natural direct and indirect effects is to identify and estimate the distribution, or aspects of the distribution, of the nested counterfactuals *Y* (a, M (a^*^)) for potentially different *a* and a^*^. As in the DAG in Figure 1, we will allow non- randomized study settings as well. The following assumptions are sufficient to identify natural direct and indirect effects on any scale from independent observations of the triplet (*C, A, Y*) [9].

### No uncontrolled confounding

Assume that the variables collected in C are sufficient for controlling confounding for a) the exposure-outcome relationship, b) the exposure-mediator relationship, and c) the mediator-outcome relationship (in [c], A is included in the set of control variables). Mathematically, the conditions are:

(1a) Y (a, M (a)) ㅛ A | C

(1b) M (a) ㅛ A | C

(1c) Y (a, m) ㅛ M | (A, C)

### Positivity

Assume that for any values of confounders, all exposure values have non-zero probability and likewise that for any values of confounders and exposure, all mediator values have non-zero probability. Mathematically, the conditions are:

(2a) P (A=a|C=c)> 0 for all a, c

(2b) P (M=m|C=c, A=a)> 0 for all a, c, m

and equivalent using densities when *A* or *M* are continuous.

### Consistency

Assume that the nested counterfactuals will actually take the observed values when the treatment and mediator are actively set to the values they would naturally have had in the absence of an intervention. Mathematically, the condition is:

(3a) P (Y (A, M)=Y)= 1 and P (M (A)=M)= 1

### Identification of natural effects

Assume that the counterfactual out come, *Y (a, m*) is independent of the counterfactual mediator, M (a^*^) when ever *a* and a^*^ are different. Mathematically, the condition is:

(4a) Y (a, m) ㅛ M (a*) | C for any m and a≠a* 

While these assumptions are structural and therefore not possible to verify using observed data alone, the identification assumption (4a) is by far the most difficult to comprehend. This assumption imposes independence between 2 distinct counterfactual worlds (because *a* and a^*^ are assumed to be different). From an applied perspective, assumption (4a) can be replaced by assuming that there are no confounders of the mediator-outcome relationship that are themselves affected by exposure. Or, perhaps more practically, one can assume that the indirect and direct effects are created by distinct and causally unrelated mechanisms.

To see why these conditions suffice, we will next derive an explicit formula for E[g (Y (a, M (a^*^))]. The arbitrary measurable function *g* : R → R is included to demonstrate that it is the full distribution of the nested counterfactual that we have identified, not only the mean. For ease of exposition only, we will assume that *C* and *M* are discrete, with state space *C* and *M*, respectively.

$$E[g(Y\;(a,\;M(a^{*}\;)))]\;=\;\sum_{c\in C}\;E[g(Y\;(a,\;M(a^{*}\;)))\;|\;C\;=\;c]P(C\;=\;c)\;=\sum_{c\in C,m\in M}E[g(Y\;(a,\;M(a*\;)))\;|\;M(a^{*}\;)\;=\;m,\;C\;=\;c]P(M(a^{*}\;)\;=\;m\;|\;C\;=\;c)P(C\;=\;c)\;=\sum_{c\in C,m\in M}E[g(Y\;(a,\;m))\;|\;M(a*\;)\;=\;m,\;C\;=\;c]P(M(a*\;)\;=\;m\;|\;C\;=\;c)P(C\;=\;c)\;\overset{i}{=}\sum_{c\in C,m\in M}E[g(Y\;(a,\;m))\;|\;C\;=\;c]P(M(a^{*}\;)\;=\;m\;|\;C\;=\;c)P(C\;=\;c)\;\;\overset{ii}{=}\sum_{c\in C,m\in M}E[g(Y\;(a,\;m))\;|\;A\;=\;a,\;M\;=\;m,\;C\;=\;c]P(M(a^{*}\;)\;=\;m\;|\;A\;=\;a^{*}\;,\;C\;=\;c)P(C\;=\;c)\;\overset{iii}{=}\sum_{c\in C,m\in M}E[g(Y\;)\;|\;A\;=\;a,\;M\;=\;m,\;C\;=\;c]P(M\;=\;m\;|\;A\;=\;a^{*}\;,\;C\;=\;c)P(C\;=\;c)\;$$

where equality *i* follows from (4a), equality *ii* from (1a-1c), and equality *iii* from (3a). The final expression only depends on the observed data and can therefore be estimated from the observed data. It appears as if the positivity assumption is not needed; however, it is precisely the positivity assumption that guarantees that, in large samples, all quantities in the final expression can be non-parametrically estimated. If one is only interested in a given function *g* and contrasts such as *E[g (Y (1, M (1)))] − E[g (Y (0, M (1)))]*, then the identification assumption can be reduced to certain no-interaction assumptions.

When *g* is reduced to the identity function, the formula is known as Pearl’s mediation formula or just the mediation formula. While the mediation formula in principle allows non-parametric estimation of any mediation analysis, it can rarely be applied directly, as one will suffer the curse of dimensionality when trying to estimate *E [g(Y) | A=a, M=m, C=c]* in all strata. As in all other branches of statistics, the curse of dimensionality is countered by introducing parametric modelling assumptions. This will be the theme of the next section.

## SECTION 3: ESTIMATING NATURAL EFFECTS MODELS

Several suggestions have been made for operationalizing the estimation of natural direct and indirect effects, such as the SPSS/SAS macros developed by Valeri & Vanderweele [10] and the R packages mediation and medflex. The topic of this section will be the class of natural effect models (NEMs) originally introduced by Lange et al. [11] and Vansterlandt et al. [12] and implemented in the R package medflex [2].

The idea underlying NEM is to phrase a mediation analysis as a multiple regression problem, thereby a) parameterizing the quantities of interest, b) allowing the choice of outcome model to follow the convention for that type of outcome (i.e., a Cox model for survival outcomes), and c) harvesting the extensive existing software implementing regression type models. In particular, a NEM is a regression model for the nested counterfactual. Expressed as a generalized linear model (GLM), it becomes:

$$g(E[Y\;(a,\;M\;(a^{*}))])=\alpha_{0}\;+\;\alpha_{1}a\;+\;\alpha_{2}a^{*}$$

If, for instance, g is the logit function, then α1 would be the marginal natural direct effect log-OR. NEMs can also be formulated conditionally on measured covariates and do not have to be only simple additive and linear effect models:

$$g(E[Y\;(a,\;M\;(a^{*}))\;|\;C=c])=ᾱW\;(a,\;a^{*},\;c)$$

where *W* is a deterministic function. The class of NEM models, along with the corresponding estimation techniques [11,12], also applies to survival models, such as the Cox model. Estimation algorithms for NEMs are all derived under the assumptions listed in the preceding section, and build on the trick that first we duplicate the original data set, then we create an artificial exposure, A^*^, which takes on different values in the 2 replications of each observation. Finally, we use an auxiliary model to link the artificial observations (i.e., those where A ≠ A^*^) to the mediators, which is either done through weighting or through imputation. Once this is done, the NEM can be estimated by simply applying standard software applied to the extended data set and using both *A* and A^*^, possibly along with *C*, as the model specification. This entire procedure has been automated and implemented in the R package medflex for any GLM type outcome model. In the following, we describe in detail how to estimate a NEM for a survival outcome with a multidimensional mediator. This is the situation we have in the illustrative case.

- Using the original data alone, fit a parametric survival model to the outcome conditioned on confounders, exposure, and mediator. This could, for instance, be a Weibull based parametric time-to-event model.
- Construct a new data set by repeating each observation in the original data set twice and including an additional variable a^*^, which is equal to the original exposure for the first replication and equal to the opposite of the actual exposure for the second replication. In addition, add an identification variable to indicate which data rows originate from the same subject.
- Use the predict functionality, possibly along with the Weibull distribution function, to impute possible survival times for the rows where A ≠ A^*^. In the imputations, the value of the exposure is set to a^*^, while mediators and confounders are set to their observed values; that is, impute values for the survival times Y_i_(a^*^, M (a)).
- Fit a Cox model to the extended data set including A, A^*^, and C, but not the mediator. The coefficient of *A* will be the natural indirect log-HR and the coefficient of a^*^ will be the natural direct log-HR.
- Repeat steps 3 and 4 ten times and combine the obtained log- HRs as with ordinary multiple imputation; that is, take the average of the log-HR estimates.
- CIs for the natural effect estimates, as well as derived quantities such as mediated proportions, can be obtained by bootstrapping, which involves repeating steps 1-5 a total of 1,000 times, each time creating a new data set by random sampling with replacement from the original data set.

## SECTION 4: ANALYSING THE ILLUSTRATIVE CASE

In our illustrative case, the outcome of interest is death from any cause during follow-up. Censoring was almost exclusively administrative, as emigration is rare in this population. The underlying time scale is in years, starting at 30 days after hospital discharge. The mediator is the 4-dimensional variable indicating whether each of the 4 recommended secondary treatments was followed.

As exposure to aspirin, P2Y12 inhibitors, statins, and β-blockers could not reasonably be said to constitute distinct causal pathways, but were highly interdependent, mediation was only assessed through the combined 4-dimensional mediator. Accordingly, the counterfactual mediator was *M (a)* є {0, 1}^4^, where *a*=0 indicates conservative invasive treatment and *a*=1 indicates early invasive treatment. The nested counterfactual was death time in years, starting 30 days after hospital discharge. To accommodate censoring, the nested counterfactual outcome technically had 2 dimensions, namely, an event time and an event indicator Y (a, M (a^*^))=((T, δ) (a, M (a^*^)). For ease of exposition, we will only refer to the underlying event time and event indicator when required by context. Our effect measure of interest was natural direct and indirect HRs decomposing the total effect, which had a HR of 0.71 (95% CI, 0.67 to 0.74). Accordingly, the final natural effects model (i.e., the one fitted in step 4 of our suggested approach) should be a Cox proportional hazard model.

Table 1 presents simple descriptive statistics of the data. Because of the very large sample size, all associations between the treatment strategy and confounders, as well as mediators, were highly significant.

**Figure 2.**
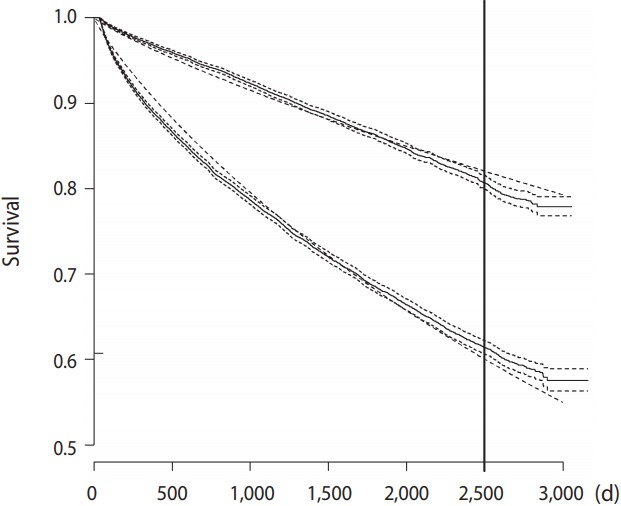
Kaplan-Meier curves (full line) along with survival curves (finely dashed lines) from a fitted parametric model with a Weibull error distribution. The lower curves are for the conservative strategy group, while the upper is for the early invasive strategy.

Figure 2 presents Kaplan-Meier curves for the 2 treatment strategy groups. It also presents the curves obtained by fitting a parametric survival model with a Weibull error distribution using only the treatment groups as covariates. It clearly shows the large differences in raw survival between the groups, and, more importantly, the figure also demonstrates that the simple 3-parameter model does a good job of capturing the distributions (Figure 2).

**Step 1:** For the actual mediation analysis, we fit a parametric survival model with a Weibull error distribution to the survival times using the treatment group, mediators, and a long list of potential confounders as explanatory variables (Appendix 1). The Appendix 2 presents both the employed R code and the full table of estimated parameters. Note that for technical reasons relating to R, it is better to use a copy of the exposure variable when fitting this model.

**Step 2:** The data set can be duplicated and the auxiliary variable inserted by copying the original data set twice. In both copies a new variable is created (called, say, exposure Star). In the first copy, the new variable is set to the values of the actual received treatment (exposure Star=exposure) while in the second copy, the new variable is set to the opposite value of the actual received treatment. Finally, the two copies are appended producing a single data set with twice as many rows as in the original data set. See the R code in Appendix 2 for coding advice.

**Step 3:** We now set the temporary exposure variable, used when fitting the imputation model in step 1, to the values in the just created exposure variable (i.e., exposure Star). As the employed survival model is parametric, we can randomly draw survival times conditional on observed mediators and the just-defined temporary exposure variable. This corresponds to randomly drawing the nested counterfactuals variables Y_a^*^,a_, where a^*^ and *a* are potentially different. To avoid extrapolating the imputation model outside what is supported by the data, any imputed survival time longer than the decided maximum follow-up (7 years in this case) will be artificially censored at the time of maximal follow-up. For the R implementation, see lines 21 to 32 of the Appendix 2.

**Step 4-5:** For each draw of the imputed nested counterfactuals, we fit a Cox model including the exposure that was actually received (the coefficient of this variable will estimate the natural indirect log-HR), the created exposure variable (the coefficient of this variable will estimate the natural direct log-HR), and all confounders. Note that the mediators are not included in this model. This is repeated 10 times, and the 10 resulting model fits are combined using standard formulas for multiple imputation. The resulting estimates are reported in Appendix 1. The fitted model is a natural effects Cox model.

**Step 6:** Finally, CIs are established by 5,000 bootstrap repetitions of steps 1-5. From the bootstrapped replications, we also directly obtain CIs for derived quantities, as the total effect (the sum of natural direct and indirect log-HRs) and the mediated proportion (natural indirect log-HR divided by total effect log-HR). The results are reported in Table 2.

From the Table 2, it is observed that after controlling for confounders, the use of early invasive treatment was associated with a reduction in 1-year mortality of 30% (OR, 0.70). The effect of early invasive treatment has 2 components: an indirect effect through secondary preventive medication, reducing risk by 10% (OR, 0.90), and the effect through all other pathways, reduces risk by a further 23%. An equivalent statement is that between a quarter and a third of the beneficial effect of early invasive treatment was achieved through the use of the 4 discharge medications. Arguably, this part of the survival gain could be achieved without adopting a full early invasive strategy, but instead by increasing the use of the 4 discharge medications to the levels seen in patients who underwent an early invasive treatment.

### R package medflex to avoid own coding

As demonstrated above and in the Appendix 2, the natural effect Cox models requires some independent coding from the researcher. This is in contrast to most other types of outcomes (continuous, binary, counts, etc.) where estimating the natural effect models has been completely automated in the R package medflex [2]. At present, the medflex package does not support survival models; however, such functionality is expected to be introduced in upcoming versions of the package. To illustrate the use of the package, we will reanalyse the illustrative case using 1-year survival, which is essentially fully observed in the data and can therefore be analysed using a natural effects logistic model, which is fully supported by the medflex package. Across the sample, we have 3,610 deaths within 1 year, corresponding to 7.3%. The medflex package will carry out the same steps as described above, but in an automated manner. Accordingly, the first step is to specify an imputation model and feed this model to the function neModel. Here, we also specify the number of mediators. The code is included below, where BinaryMort is the outcome and dhrkag3 the exposure. The mediators are asatreat30, adptreat30, statintreat30, and betatreat30 and the number of mediators is specified using the nMed argument. All other variables are confounders; see Appendix 1 for definitions of the variables.

fitAux ← glm (BinaryMort ~ dhrkag3 + asatreat30 + adptreat30
+ statintreat30 + betatreat30 + i_alder + factor (sex)
+ factor (indkgrp) + factor (uddankat) + boralene + factor (fi_aar)
+ mi + card + cochf + puled + shock + cervas + mal + diabet
+ arf + crf + anemi + pneumoni + sepsis + klap + bleed
+ Antihyp_12mb + Lipidlow_12mb + ASA_12mb + VitKant_12mb
+ Diureti_loop_12mb + COPD_12mb + tidl_reva, data=workData, family=“binomial”)
extendedData ← neImpute(fitAux, nMed=4)

The last step is then to specify the natural effects model within the neModel function and extract the estimates for natural direct and indirect effects. The code is presented below.

fit NEM_binaryOutcome ← neModel (BinaryMort ~ dhrkag30 + dhrkag31
+ i_alder + factor (sex) + factor (indkgrp) + factor (uddankat)
+ boralene + factor (fi_aar) + mi + card + cochf + puled + shock
+ cervas + mal + diabet + arf + crf + anemi + pneumoni + sepsis
+ klap + bleed + Antihyp_12mb + Lipidlow_12mb + ASA_12mb
+ VitKant_12mb + Diureti_loop_12mb + COPD_12mb + tidl_reva, expData=extendedData, family=“binomial”, se=“robust”

Summary (neEffdecomp (fitNEM_binaryOutcome))

The neImpute function creates the 2 new auxiliary exposure variables dhrkag30 and dhrkag31, which correspond to the natural direct and indirect effects, respectively. As the natural effects model is in this case a logistic regression, the estimates are presented as ORs in the Table 3.

From the Table 3, it is observed that the mediated proportion is similar to what was found in the Cox-based analysis, but with wider CIs. As we are using fewer events in the analysis, the wider CIs are to be expected. The effect estimates are similar to the Cox-based analysis, but numerically smaller; however, as one is a HR and the other is an OR, they cannot be directly compared.

## SECTION 5: NEW CHALLENGES WITHIN MEDIATION ANALYSIS

As outlined in the preceding sections, mediation analysis with a single well-defined mediator (possibly multi-dimensional) and associated simple causal structure has by now been very well researched. This includes theoretical considerations and software implementations. For the applied researcher, a review of existing software solutions written by Starkopf et al. [13] is under review and available as a working paper upon request. On the purely applied side, we still need to see more applications, mainly to address subject matter problems, but also to establish common best practices for conducting mediation analyses.

This by no means implies that there are no unsolved methodological questions within mediation analysis. We see some of the most pressing problems as:

First, how to handle measurement error for the mediator. Currently, the best suggestion is to conduct sensitivity analyses assessing the potential impact of such measurement errors. This is of course good, but it would be more fruitful to have methods that could handle measurement errors directly. Mplus has capabilities in this direction [14], but they come at the cost of numerous parametric assumptions, and worse, a reduced causal interpretation, because effects are expressed on a latent and somewhat arbitrarily defined scale. Second, further methods to handle causally ordered mediators and/or mediators measured repeatedly over time. Important work in this regard was recently published [15]. Moreover, in the context of a survival outcome, the problem is further complicated by the fact that death has a truncation effect on the mediator process [16].

Third, existing software for mediation analysis should be extended to make it easier to conduct sensitivity analyses.

## CONCLUSION

It is our hope that this tutorial has shown the potential of mediation analyses in discovering the causal mechanisms underlying a given cause-and-effect relation, and has demonstrated the relative ease with which mediation analyses can be conducted using standard software.

## Figures and Tables

**Table 1.** Descriptive statistics

	Conservative	Early invasive	p-value
n	26,858	22,782	
Mean age (yr)	69.0	63.0	<0.001
Male (%)	59.4	70.7	<0.001

**Table 2.** Summary of mediation analysis

	HR	95% CI	
		Lower limit	Upper limit
Effect^1^			
Natural indirect	0.90	0.88	0.92
Natural direct	0.77	0.76	0.79
Total	0.70	0.69	0.70
Mediated proportion	0.30	0.25	0.34

HR, hazard ratio; CI, confidence interval.

1 The effects are HRs for all-cause mortality except for the mediated proportion. These results are based on a natural effects Cox model conditional on all recorded baseline confounders.

**Table 3.** Natural direct and indirect ORs when only looking at 1-year survival

	OR	95% CI	
		Lower limit	Upper limit
Effect			
Natural indirect	0.84	0.78	0.89
Natural direct	0.66	0.62	0.70
Total	0.55	0.51	0.60
Mediated proportion	0.30	0.22	0.38

OR, odds ratio; CI, confidence interval.

## Appendix 1. Full model fits

When estimating the natural effect Cox model in the illustrative case, a Weibull parametric survival model was used as an intermediate step in order be able to impute the nested counterfactual. The model contains exposure, mediators, and all considered confounders.

After imputation, a natural effects Cox model can be estimated by fitting a Cox model to the extended data set. The table below presents estimates for a single imputation.

**Table S1a.** Model t for Weibul based parametric survival model

Variable (in R notation)	Estimate	SE	p-value
(Intercept)	13.302	0.101	0.000
dhrkag3TEMP	0.250	0.024	0.000
asa_treat30	0.080	0.015	0.000
adp_treat30	0.095	0.011	0.000
statin_treat30	0.204	0.013	0.000
beta_treat30	0.098	0.012	0.000
i_alder	-0.064	0.001	0.000
factor(sex)2	0.232	0.021	0.000
factor(indkgrp)2	0.159	0.028	0.000
factor(indkgrp)3	0.316	0.038	0.000
factor(uddankat)2	0.030	0.022	0.165
factor(uddankat)3	0.146	0.034	0.000
boralene	-0.155	0.021	0.000
factor(fi_aar)2006	0.003	0.029	0.918
factor(fi_aar)2007	-0.027	0.032	0.399
factor(fi_aar)2008	-0.146	0.038	0.000
factor(fi_aar)2009	-0.209	0.042	0.000
factor(fi_aar)2010	-0.173	0.048	0.000
factor(fi_aar)2011	-0.280	0.055	0.000
mi	-0.598	0.032	0.000
card	-0.026	0.027	0.339
cochf	-0.283	0.026	0.000
puled	-0.234	0.077	0.003
shock	-0.107	0.170	0.530
cervas	-0.341	0.034	0.000
mal	-0.985	0.039	0.000
diabet	-0.388	0.036	0.000
arf	-0.405	0.066	0.000
crf	-0.395	0.051	0.000
anemi	-0.285	0.040	0.000
pneumoni	-0.380	0.028	0.000
sepsis	-0.155	0.074	0.036
klap	-0.260	0.037	0.000
bleed	-0.102	0.048	0.034
Antihyp_12mb	-0.021	0.022	0.352
Lipidlow_12mb	0.093	0.024	0.000
ASA_12mb	-0.101	0.022	0.000
VitKant_12mb	-0.048	0.037	0.188
Diureti_loop_12mb	-0.442	0.023	0.000
COPD_12mb	-0.362	0.023	0.000
tidl_reva	0.278	0.064	0.000
Log(scale)	-0.022	0.009	0.012

**Table S1b.** Full model t for natural eect model based on a single imputation

Description	R variable names	log-HR	SE	p-value
Indirect	dhrkag3	-0.10	0.01	0.00
Direct	dhrkag3STAR	-0.26	0.01	0.00
Age	i_alder	0.07	0.00	0.00
Gender (female)	sex2	-0.21	0.01	0.00
Income (middle)	factor(indkgrp)2	-0.16	0.02	0.00
Income (high)	factor(indkgrp)3	-0.32	0.03	0.00
Education (middle)	factor(uddankat)2	-0.05	0.01	0.00
Education (high)	factor(uddankat)3	-0.12	0.02	0.00
Live alone	boralene	0.17	0.01	0.00
Year (2006)	factor(fi_aar)2006	-0.03	0.02	0.19
Year (2007)	factor(fi_aar)2007	0.00	0.02	0.92
Year (2008)	factor(fi_aar)2008	0.14	0.03	0.00
Year (2009)	factor(fi_aar)2009	0.20	0.03	0.00
Year (2010)	factor(fi_aar)2010	0.16	0.03	0.00
Year (2011)	factor(fi_aar)2011	0.25	0.03	0.00
Myocardial infarction	mi	0.49	0.02	0.00
Cardiac arrhythmia	card	0.05	0.02	0.01
chronic obstructive pulmonary disease	cochf	0.30	0.02	0.00
Pulmonary oedema	puled	0.30	0.06	0.00
Cardiogenic shock	shock	0.22	0.11	0.05
Cerebrovascular disease	cervas	0.37	0.02	0.00
Diabetes with complications	diabet	0.36	0.03	0.00
acute renal failure	arf	0.44	0.05	0.00
Chronic renal failure	crf	0.44	0.04	0.00
Anaemia	anemi	0.34	0.03	0.00
Pneumonia	pneumoni	0.46	0.02	0.00
Sepsis	sepsis	0.20	0.05	0.00
Valvular heart disease	klap	0.26	0.03	0.00
Prior in-hospital bleeding	bleed	0.17	0.03	0.00
Use of antihyp. medication last 12M	Antihyp_12mb	0.06	0.01	0.00
Use of lipid-lowering drugs last 12M	Lipidlow_12mb	-0.04	0.02	0.02
Asprin	ASA_12mb	0.16	0.01	0.00
Use of vitamin K antagonists last 12M	VitKant_12mb	0.09	0.02	0.00
Use of glucose-lowering drugs lst 12M??	Diureti_loop_12mb	0.50	0.02	0.00
Use of loop diuretics or COPD last 12M	COPD_12mb	0.39	0.02	0.00
Prior revascularization	tidl_reva	-0.24	0.04	0.00

## Appendix 2. Employed R code

The full R code used for estimating the natural effect Cox model is presented below.

## References

[1] Baron RM, Kenny DA (1986). The moderator-mediator variable distinction in social psychological research: conceptual, strategic, and statistical considerations. J Pers Soc Psychol.

[2] Steen J, Loeys T, Moerkerke B, Vansteelandt S (2017). Medflex: an R package for flexible mediation analysis using natural effect models. J Stat Softw.

[3] Hansen KW, Sorensen R, Madsen M, Madsen JK, Jensen JS, von Kappelgaard LM (2015). Effectiveness of an early versus a conservative invasive treatment strategy in acute coronary syndromes: a nationwide cohort study. Ann Intern Med.

[4] Roffi M, Patrono C, Collet JP, Mueller C, Valgimigli M, Andreotti F (2016). 2015 ESC guidelines for the management of acute coronary syndromes in patients presenting without persistent ST-segment elevation: Task Force for the Management of Acute Coronary Syndromes in Patients Presenting without Persistent ST-Segment Elevation of the European Society of Cardiology (ESC). Eur Heart J.

[5] Hansen KW, Sorensen R, Madsen M, Madsen JK, Jensen JS, von Kappelgaard LM (2015). Effectiveness of an early versus a conservative invasive treatment strategy in acute coronary syndromes: a nationwide cohort study. Ann Intern Med.

[6] Pearl J (2009). Causality: models, reasoning, and inference.

[7] Hernán MA, Robins JM (2012). Causal inference.

[8] Pearl J, Jack Breese J, Koller D (2001). Direct and indirect effects. American Association for Artificial Intelligence. Uncertainty in artificial intelligence. Proceedings of the Seventeenth Conference.

[9] Vanderweele TJ (2015). Explanation in-causal inference: methods for mediation and interaction.

[10] Valeri L, Vanderweele TJ (2013). Mediation analysis allowing for exposure-mediator interactions and causal interpretation: theoretical assumptions and implementation with SAS and SPSS macros. Psychol Methods.

[11] Lange T, Vansteelandt S, Bekaert M (2012). A simple unified approach for estimating natural direct and indirect effects. Am J Epidemiol.

[12] Vansteelandt S, Bekaert M, Lange T (2012). Imputation strategies for the estimation of natural direct and indirect effects. Epidemiol Methods.

[13] Starkopf L, Andersen MP, Gerds TA, Torp-Pedersen C, Lange T (2017). Comparison of five software solutions to mediation analysis. https://ifsv.sund.ku.dk/biostat/annualreport/images/0/0a/Research_Report_17-01.pdf.

[14] Muthen B, Muthen LK, Asparouhov T (2016). Regression and mediation analysis using Mplus.

[15] Steen J, Loeys T, Moerkerke B, Vansteelandt S (2017). Flexible mediation analysis with multiple mediators. Am J Epidemiol.

[16] Strohmaier S, Haase N, Wetterslev J, Lange T (2017). A simple to implement algorithm for natural direct and indirect effects in survival studies with a repeatedly measured mediator. http://www.stat-center.pku.edu.cn/Stat/Index/research_show/id/227.

