# Supplementary material for: Applied mediation analyses: a review and tutorial
Source: Epidemiol Health. 2017 Aug 6;39:e2017035. doi: 10.4178/epih.e2017035 (PMC5723912; doi:10.4178/epih.e2017035)
Supplement: Supplementary file 1 [file epih-39-e2017035-app2.pdf]

Appendix 2. Employed R code

The full R code used for estimating the natural effect Cox model is presented below.

```
# load data and libraries
library(medflex)
library(mice)
library(survival)
workData <- read.csv("full_data.csv")
maxFollowUpTimeTemp <- 7*365.25

### code to do full mediation analysis
### Define main function
fitNEM <- function(workData, maxFollowUpTimeTemp)
{
  # fit mediation model
  workData$dhrkag3TEMP <- workData$dhrkag3
  fitImp <- survreg(Surv(obs_dod, dod) ~ dhrkag3TEMP + asa_treat30 + adp_treat30 + statin_treat30 + beta_treat30
                     + i_alder + factor(sex) + factor(indkgrp) + factor(uddankat) + boralene + factor(fi_aar) + mi + card
                     + cochf + puled + shock + cervas + mal + diabet + arf + crf + anemi + pneumoni + sepsis
                     + klap + bleed + Antihyp_12mb + Lipidlow_12mb + ASA_12mb + VitKant_12mb + Diureti_loop_12mb
                     + COPD_12mb + tidl_reva,
                     data=workData)

  # do dataset expansion and imputation
  tempData1 <- workData
  tempData1$dhrkag3STAR <- tempData1$dhrkag3
  tempData2 <- workData
  tempData2$dhrkag3STAR <- 1-tempData2$dhrkag3
  tempData2$dhrkag3TEMP <- tempData2$dhrkag3STAR
  linPredTemp <- predict(fitImp, newdata = tempData2, type="linear")
  simDodTimesTemp <- rweibull(nrow(tempData2), shape = 1/fitImp$scale, exp(linPredTemp))
  tempData2$dod <- 1*(simDodTimesTemp<maxFollowUpTimeTemp)
  tempData2$obs_dod <- simDodTimesTemp*(simDodTimesTemp<maxFollowUpTimeTemp) + maxFollowUpTimeTemp*(simDodTimesTemp
```

```r
    >=maxFollowUpTimeTemp)

  expData <- rbind(tempData1, tempData2)

  # fit natural effects model
  fitNEM <- coxph(Surv(obs_dod, dod) ~ dhrkag3 + dhrkag3STAR
                  + i_alder + sex + factor(indkgrp) + factor(uddankat) + boralene + factor(fi_aar) + mi + card
                  + cochf + puled + shock + cervas + mal + diabet + arf + crf + anemi + pneumoni + sepsis
                  + klap + bleed + Antihyp_12mb + Lipidlow_12mb + ASA_12mb + VitKant_12mb + Diureti_loop_12mb
                  + COPD_12mb + tidl_reva,
                  data=expData)
  # summary(fitNEM)
  # return(fitNEM)
  return(summary(fitNEM)$coefficients)
}

#### Get par estimates
tempFitNEM <- fitNEM(workData, maxFollowUpTimeTemp=maxFollowUpTimeTemp)
tempFitNEM

Nimp <- 10
outTable <- array(NA, dim=c(dim(tempFitNEM),Nimp))
for(j in 1:Nimp)
{
  outTable[ , ,j] <- fitNEM(workData, maxFollowUpTimeTemp=maxFollowUpTimeTemp)
}

library(Amelia)
temp <- mi.meld(q = outTable[,1,], se = outTable[ , 2, ], byrow = F)
tempOut <- tempFitNEM[,1:2]
tempOut[,1] <- temp$q.mi
tempOut[,2] <- temp$se.mi
tempOut

### get bootstrap SDs
G <- 10^3
```

```
outputObj <- array(NA, dim = c(dim(tempFitNEM),G))
for(j in 1:G)
{
  tempData <- workData[sample(1:nrow(workData)), ]
  temp <- try(fitNEM(tempData, maxFollowUpTimeTemp), silent=TRUE
      )
  if(class(temp)!="try-error") outputObj[, ,j] <- temp
  rm(tempData, temp)
  cat(paste("\n  Itteration ", j," of ", G, " comp."))
  save(outputObj, file="outputObj.Rdata")
}

outTable <- tempFitNEM[,1:2]
outTable[,1] <- apply(outputObj[,1,],1, mean,  na.rm=T)
outTable[,2] <- apply(outputObj[,1,],1, sd,  na.rm=T)
write.csv(outTable, file="fitNEMbootstrap.csv")

IE <- outputObj[1,1, ]
DE <- outputObj[2,1, ]
TE <- IE+ DE
Q <- IE/TE

outTable <- matrix(NA, nrow=4, ncol=3)
outTable[1, ] <- c(mean(IE), quantile(IE, c(.025, .975)))
outTable[2, ] <- c(mean(DE), quantile(DE, c(.025, .975)))
outTable[3, ] <- c(mean(TE), quantile(TE, c(.025, .975)))
outTable[4, ] <- c(mean(Q), quantile(Q, c(.025, .975)))
rownames(outTable) <- c("IE", "DE", "TE", "Q")
outTable
```
